# Comment on “Change in Physical Activity and Its Association With Decline in Kidney Function: A UK Biobank‐Based Cohort Study” by Liu et al.—The Authors' Reply

**DOI:** 10.1002/jcsm.13765

**Published:** 2025-03-05

**Authors:** Qiaoling Liu, Carlos Celis‐Morales, Jennifer S. Lees, Naveed Sattar, Frederick K. Ho, Jill P. Pell, Patrick B. Mark, Paul Welsh

**Affiliations:** ^1^ School of Cardiovascular and Metabolic Health University of Glasgow Glasgow UK; ^2^ Human Performance Lab, Education, Physical Activity and Health Research Unit University Católica del Maule Talca Chile; ^3^ School of Health and Wellbeing University of Glasgow Glasgow UK

Dear Dr. Wang and colleagues,

We are grateful for the interest of Dr Wang et al. [1] in our study. Their letter raises several important points, and we are pleased to have the opportunity to address them.

As Wang G et al. note, the UK Biobank does include accelerometer‐measured physical activity data. However, these measurements were collected in a subset of participants (< 100 000) at a single time point, 8–10 years after the baseline assessment. Unfortunately, biomarkers were not assessed at the time when the accelerometer data were collected. Consequently, it is not feasible to evaluate changes in physical activity over time—our study's primary exposure of interest—or to link these accelerometer‐based physical activity measures with kidney function outcomes. We fully agree with Wang G et al.'s observation that self‐reported physical activity data inherently cause some misclassification. As discussed in the manuscript, recall bias can be bidirectional and any misclassification would likely be nondifferential and expected to underestimate the magnitude of the effect size. We have acknowledged this potential bias as the first limitation [2]. We would also point out that the associations of self‐reported and objectively measured physical activity with health outcomes are generally concordant in UK Biobank [3, 4, 5]. Further research involving repeated assessments of accelerometer‐based physical activity would greatly improve our understanding of how activity patterns influence kidney function. However, large cohort studies with data collected at multiple time points are not yet available.

Regarding mGFR, Porrini et al. (2019) reviewed that eGFR can deviate from mGFR by around 30% [6]. However, current mGFR measurement methods—whether using inulin, iohexol, or other filtration markers—are complex, expensive and simply not feasible in very large cohorts. Many institutions, including KDIGO, recommend using eGFR in most cases, leaving mGFR for specific clinical scenarios [7]. Given the large sample size in our study and the fact that participants do not have known kidney disease, we believe that eGFR is suitable for our research needs. To mitigate known limitations of eGFR, we used creatinine‐based, cystatin C‐based and creatinine + cystatin C‐based eGFR estimates, accepting that some interpretation is needed to account for the non‐GFR determinants of creatinine and cystatin C.

We agree that diet can have an impact on serum creatinine, which was the purpose of our study also reporting eGFR_cysC_. To our knowledge, there is no evidence suggesting that diet directly impacts cystatin‐C [8]. However, our study accounted for BMI, which serves as a reasonable proxy for overall diet quality. Additionally, we adjusted our analyses for inflammation, including CRP, to ensure robustness.

We understand Dr Wang et al.'s suggestion of additional focus on patients with CKD. We recognise the wider research on the effect of exercise on CKD. For instance, a meta‐analysis of 12 RCTs demonstrated that regular aerobic exercise can improve estimated glomerular filtration rate, serum creatinine, 24‐h urine protein levels and blood urea nitrogen in CKD patients [9]. Studies on the general population, especially large‐scale studies, focusing on kidney function are less common. From a preventive medicine perspective, if individuals without kidney disease can enjoy renal benefits from increasing physical activity, this might, in the long run, reduce the incidence of chronic diseases. This motif is central to our study.

Finally, we agree with Wang et al. that further evidence is required. Randomized controlled trials are the gold standard for causal inference, although whether these are feasible at scale requires careful consideration.
